# Encapsulation of Lutein via Microfluidic Technology: Evaluation of Stability and In Vitro Bioaccessibility

**DOI:** 10.3390/foods10112646

**Published:** 2021-11-01

**Authors:** Yuanhang Yao, Jiaxing Jansen Lin, Xin Yi Jolene Chee, Mei Hui Liu, Saif A. Khan, Jung Eun Kim

**Affiliations:** 1 Department of Food Science and Technology, National University of Singapore, Singapore 117543, Singapore; yuanhang@u.nus.edu (Y.Y.); jolene_chee@sics.a-star.edu.sg (X.Y.J.C.); fstlmh@nus.edu.sg (M.H.L.); 2 Department of Chemical and Biomolecular Engineering, National University of Singapore, Singapore 117585, Singapore; mdclinj@nus.edu.sg (J.J.L.); saifkhan@nus.edu.sg (S.A.K.)

**Keywords:** lutein, stability, bioaccessibility, encapsulation, microfluidics

## Abstract

Inadequate intake of lutein is relevant to a higher risk of age-related eye diseases. However, lutein has been barely incorporated into foods efficiently because it is prone to degradation and is poorly bioaccessible in the gastrointestinal tract. Microfluidics, a novel food processing technology that can control fluid flows at the microscale, can enable the efficient encapsulation of bioactive compounds by fabricating suitable delivery structures. Hence, the present study aimed to evaluate the stability and the bioaccessibility of lutein that is encapsulated in a new noodle-like product made via microfluidic technology. Two types of oils (safflower oil (SO) and olive oil (OL)) were selected as a delivery vehicle for lutein, and two customized microfluidic devices (co-flow and combination-flow) were used. Lutein encapsulation was created by the following: (i) co-flow + SO, (ii) co-flow + OL, (iii) combination-flow + SO, and (iv) combination-flow + OL. The initial encapsulation of lutein in the noodle-like product was achieved at 86.0 ± 2.7%. Although lutein’s stability experienced a decreasing trend, the retention of lutein was maintained above 60% for up to seven days of storage. The two types of device did not result in a difference in lutein bioaccessibility (co-flow: 3.1 ± 0.5%; combination-flow: 3.6 ± 0.6%) and SO and OL also showed no difference in lutein bioaccessibility (SO: 3.4 ± 0.8%; OL: 3.3 ± 0.4%). These results suggest that the types of oil and device do not affect the lutein bioaccessibility. Findings from this study may provide scientific insights into emulsion-based delivery systems that employ microfluidics for the encapsulation of bioactive compounds into foods.

## 1. Introduction

Carotenoids are a family of terpenoid pigments rich in fruits and vegetables and are related to several potential health benefits because of their antioxidant and anti-inflammatory properties [1,2,3,4,5]. Lutein is one of the main carotenoids that can selectively accumulate in the eye, macula and retina in particular, and is known for eye protection effects, especially against photoinduced damage [6,7,8]. This is mainly because lutein is capable of quenching singlet oxygen and other reactive oxygen species and absorbing blue light [6]. Abundant epidemiological evidence has suggested that lutein intake is positively correlated with a lower risk of age-related macular degeneration and cataracts [9,10,11]. Nevertheless, lutein can only be obtained from diets, which is often insufficient due to the limited consumption of fruits and vegetables. Additionally, the biological activity of lutein is highly dependent on its gastrointestinal absorption, which may be hindered mainly as a consequence of its physicochemical properties [12,13]. 

One approach to overcome the challenges of insufficient lutein intake is to develop lutein-enriched staple foods, thus delivering lutein to humans regularly and continuously. However, merely incorporating lutein as an ingredient into foods and beverages hardly exerts the nutritional value of lutein due to its poor solubility and the fact that lutein is prone to oxidative degradation [13,14]. Encapsulation technology has shown to be an attractive strategy to entrap the bioactive compounds within a carrier, which can improve their stability during food processing, storage and gastrointestinal absorption [15,16,17,18]. Microfluidics has become a trending topic in innovative food processing in recent years, with nutrients encapsulation being an emerging application of the microfluidic technique [19]. Microfluidics particularly focuses on accurate control over minute volumes of fluids within a system of microchannels [20,21]. This makes it possible to alter the way of working with dispersed food systems, and inherently manipulate structures at a micro-level [19].

Lutein has to be incorporated into mixed micelles for gastrointestinal absorption due to its hydrophobic property. Bioaccessibility describes the fraction of lutein solubilized in the mixed micelles and is usually determined in vitro via a simulated gastrointestinal digestion model [22]. Several lines of studies have indicated that fat-soluble carotenoids such as lutein, when dispersed in dietary oils, obtain greater bioaccessibility than when they are consumed alone [23,24,25]. This is because oils work as a delivery vehicle for these hydrophobic nutrients: The hydrolysis products of oils—free fatty acids and monoglycerides—together with phospholipids, bile salts and cholesterol, form the mixed micelles in the aqueous digestion fluid [26]; the formation of the mixed micelles facilitates the solubilization of hydrophobic lutein in mixed micelles and makes lutein become accessible during digestion [23,27]. Moreover, several studies have reported that the emulsion-based delivery system shows desired properties such as alleviating the degradation of bioactive compounds, improving the efficiency in micellization and promoting the digestive enzyme activity [28,29,30]. 

However, considering microfluidics is a relatively innovative technology, especially in “foods” area, limited studies have investigated its potential in nutrient encapsulation combined with an emulsion-based delivery system. Additionally, the evidence of applying this microfluidic technique to encapsulate carotenoids into foods is lacking. Therefore, this study aimed to encapsulate lutein into a staple food noodle using excipient emulsions via microfluidics-based continuous extrusion technique, and to assess the stability and bioaccessibility of lutein with different microfluidic assemblies and different types of oils.

## 2. Materials and Methods

### 2.1. Materials

Food-grade sodium alginate and calcium chloride (CaCl_2_) were purchased from a local shop (Phoon Huat, Singapore). Soy protein isolate (SPI, New Fujipro SEH; Fuji Oil Co., Ltd., Tokyo, Japan) formulation was generously sponsored by Fuji Oil Holdings Inc. (Izumisano-shi, Japan), and its protein content was about 90%. FloraGlo lutein was sponsored by DSM company (Dutch State Mines, DU, Heerlen, The Netherlands) and was known to be extracted from *Tagetes erecta*. Safflower oil (SO) was purchased from iHerb (Eden Foods, Clinton, Michigan, US) and olive oil (OL) was purchased from the local supermarket (NTUC FairPrice, Singapore). Pure lutein standards, amylase (10 U/mg), pepsin (695 U/mg), pancreatin (P7545, 8 X USP) and bile salt were purchased from Sigma Aldrich (St. Louis, MI, USA). All used chemicals were of analytical-grade and all solvents for lutein extraction were of high-performance liquid chromatography (HPLC)-grade.

### 2.2. Sample Preparation

Sodium alginate was blended with deionized water to form a 2% (*w*/*v*) solution, and was centrifuged at 19,802× *g*, 20 °C for 15 min. SPI was blended with deionized water to form a 12% (*w*/*v*) solution at room temperature and underwent ultrasonic degassing for 20 min. The pH of the SPI solution was determined to be 7.51. The viscosity of the prepared sodium alginate solution and SPI solution was determined to be 2630 and 10,590 cP, respectively, at 20 rpm speed. CaCl_2_ solution (3% *w*/*v*) was prepared by dissolving CaCl_2_ in deionized water. Lutein fortified OL (0.5% *w*/*v*) or SO (0.5% *w*/*v*) was added in the SPI solution to form the emulsion by using the high shear mixer (Silverson L4RT, US) at 8000 rpm for 7 min, and the container was placed under the ice bath to prevent the excessive heat. The ratio of the lutein fortified oil to SPI solution was 10:1 (*v*/*v*).

### 2.3. Assembly of Microfluidic Devices

Assembly of microfluidic devices was performed based on the methods disclosed in a patent (No. 10201906256W). The flow rate was set at 3000 μL/min for sodium alginate solution and 500 μL/min for SPI and lutein-fortified oil emulsion for the co-flow device, while it was set separately at 454.5 μL/min and 45.5 μL/min for the SPI solution and lutein-fortified oil for the combination of co-flow and flow-focusing device (namely combination-flow). 

**Figure 1:**
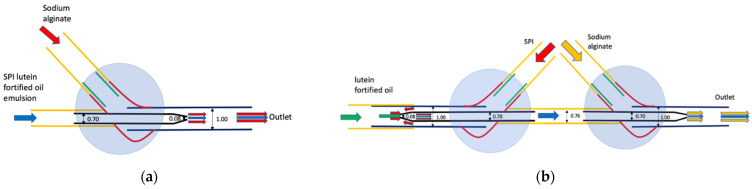
Schematic diagram of microfluidic devices (**a**) Co-flow; (**b**) Combination-flow. SPI: Soy protein isolate. Critical channel diameters are measured in millimeters.

Schematic diagrams of two different microfluidic devices are shown in Figure 1. The co-flow device (Figure 1a) is made up of an inner circular capillary that is fitted in an outer square capillary, with both the inner (SPI and lutein fortified oil emulsion) and outer (sodium alginate) fluids flowing in the same direction. Flow-focusing is similar to the co-flow device, whereby the inner (lutein fortified oil) and outer (SPI) fluids are introduced in opposing directions to each other before exiting through the same capillary. Combination-flow (Figure 1b) consists of the flow-focusing on the left part and co-flow on the right part. The mixture of lutein-fortified oil and SPI is extruded first with flow-focusing and then follows the fate of the co-flow; flowing through the inner capillary with the same direction as sodium alginate, which flows in the outer capillary. The material composition of extrudates are theoretically the same for both two devices. The extruded noodle-like samples were subjected to a CaCl_2_ bath for 30 min for ionic gelation and were subsequently heated in a deionized water bath (80–85 °C) for 6 min for thermal gelation. Afterwards, all noodle samples were kept in water as dehydration may occur due to the diffusion of saturated water vapor at the surface [31]. All the above procedures were done under dull red light to minimize the photodecomposition of lutein.

### 2.4. In Vitro Digestion

The microfluidic noodle was cut into < 2 mm pieces before being subjected to the simulated in vitro digestion. The protocol for digestion was referred from the INFOGEST method with minor modification [32]. All simulated saliva fluid (SSF), simulated gastric fluid (SGF) and simulated intestinal fluid (SIF) solutions were prepared according to the INFOGEST method. Specifically, SSF was comprised of 15.1 mmol/L potassium chloride (KCL), 3.7 mmol/L potassium dihydrogen phosphate (KH_2_PO_4_), 13.6 mmol/L sodium bicarbonate (NaHCO_3_), 0.15 mmol/L magnesium chloride hexahydrate (MgCl_2_(H_2_O)_6_) and 0.06 mmol/L ammonium carbonate ((NH_4_)_2_CO_3_); SGF was comprised of 6.9 mmol/L KCL, 0.9 mmol/L KH_2_PO_4_, 25 mmol/L NaHCO_3_, 47.2 mmol/L NaCl, 0.1 mmol/L MgCl_2_(H_2_O)_6_ and 0.5 mmol/L (NH_4_)_2_CO_3_; SIF was comprised of 6.8 mmol/L KCL, 0.8 mmol/L KH_2_PO_4_, 85 mmol/L NaHCO_3_, 38.4 mmol/L NaCl and 0.33 mmol/L MgCl_2_(H_2_O)_6_. To start, 5 g of cut noodle samples were mixed with 3.5 mL SSF, 0.5 mL salivary a-amylase (1500 U/mL) solution, 25 uL CaCl_2_ (0.3 M) and 975 uL deionized water, and were incubated in the water bath at 37 °C, 85 rpm for 2 min. Gastric digestion was started with the oral digesta being acidified, by adding 1 M hydrogen chloride (HCL) (~0.2 mL) to adjust the pH to reach 3.0. It was subsequently mixed with 7.5 mL SGF, 5 uL CaCl_2_ (0.3 M), 1.6 mL pepsin (25,000 U/mL) solution and 695 uL deionized water, and was incubated in the water bath at 37 °C, 85 rpm for 1.5 h. Afterwards, to simulate the intestinal digestion, 1 M sodium hydroxide (NaOH) (~0.15 mL) was used to adjust the pH to 7.0, and then was mixed with 11 mL SIF, 5 mL pancreatin solution (800 U/mL), 2.5 mL bile (10 mM), 40 uL CaCl_2_ (0.3 M) and 1.31 mL deionized water, and was incubated in the water bath at 37 °C, 85 rpm for 2.5 h. Right after the final stage of the simulated gastrointestinal digestion was completed, raw digesta was centrifuged at 19,802× *g*, 20 °C for 45 min. Given that large particles are unable to pass through the mucus layer and be taken up by epithelium cells [33], the aqueous supernatant after filtration (pore size 0.45 μm) was assumed to be comprised of mixed micelles where the lutein solubilized [34]. We considered the lutein solubilized in the micellar fractions as the bioaccessible lutein. During the entire digestion procedure, all the samples were kept in the amber color tubes or the containers were covered with aluminum foil to minimize the photodecomposition of lutein.

### 2.5. Extraction and Quantification of Lutein

Lutein in digesta, micelle fraction and homogenate were extracted and analyzed as previously reported [35]. Briefly, digesta, micellar fraction or homogenate was extracted with acetone:petroleum ether (1:1, *v*/*v*, second and third times was extracted with petroleum ether alone), vortexed for 2 min and was centrifuged for 10 min at 19,802× *g*, 20 °C. The supernatant layer was collected and the above extraction was repeated three times. All the supernatant layers were combined, and then it was evaporated by nitrogen gas. The final samples were reconstituted in methanol:methyl tert-butyl ether (MTBE) (1:1, *v*/*v*) and were filtered through a 0.45 μm filter. The extraction procedure was fully carried out under dull red light, and 0.1% butylated hydroxytoluene (*w*/*v*) was added in the extraction solvents to minimize lutein degradation.

Lutein was detected by the HPLC (Waters, US) at 4 °C at the wavelength of 450 nm with a YMC carotenoid C30 column, 250 mm × 4.6 mm ID (YMC, Japan), that has been reported previously [35]. The mobile phases were comprised of methanol:MTBE:water (A, 81:15:4, *v*/*v*/*v*) and methanol:MTBE:water (B, 9:87:4, *v*/*v*/*v*). The gradient program was carried out as follows: an initial condition of eluent A:B was 100:0 (%), then there was a linear increase till A:B was 81:19 (%) at 3 min, followed by an A:B of 47:53 (%) at 25 min, and then a rapid increase till A:B was 0:100 (%) at 27 min, held for 10 min and finally back to the initial condition in 3 min, allowing for a 10 min hold as re-equilibration. The flow rate was set as 1 mL/min and the injection volume was 80 μL.

### 2.6. Optical Microscopy

Images of microfluidic noodle with two types of devices (co-flow and combination-flow) were obtained using a microscope digital camera DP74 mounted on an Olympus BX51 light microscope. The images were viewed under 4× magnification.

### 2.7. Storage Stability

The stability of lutein was represented by the retention of lutein in the microfluidic noodle at each storage day 1, 2, 3, 4, 5, 6 and 7 under 4 °C as compared to the initial added lutein content. The storage stability was calculated as follows:

(1) $$Stability\left(\%\right)=\frac{100\times C_{sample}}{C_{intial}}$$

where $C_{sample}$ is the remaining lutein content in the microfluidic noodle samples at each storage day, and $C_{intial}$ corresponds to the initial added lutein content.

### 2.8. Bioaccessibility, Release and Micellarization of Lutein

The fraction of lutein solubilized in the mixed micelles phase after passing through the simulated in vitro digestion was taken to be bioaccessibility and was calculated as follows:

(2) $$Bioaccessibility\left(\%\right)=\frac{100\times C_{micelles}}{C_{intial}}$$

The release rate was determined as the lutein content in the digesta released from the initial food matrix and was calculated as follows:

(3) $$Release\left(\%\right)=\frac{100\times C_{digesta}}{C_{intial}}$$

The micellarization rate was determined as transfer of lutein from the digesta to the mixed micelles and was calculated as follows:

(4) $$Micellarization\left(\%\right)=\frac{100\times C_{micelles}}{C_{digesta}}$$

where $C_{micelles}$ is lutein content in the micellar fraction, $C_{digesta}$ is lutein content in the digesta, and $C_{intial}$ is the initial added lutein content in the microfluidic noodle. All the determinations of lutein bioaccessibility, release and micellarization were conducted on day 1.

**Figure 2:**
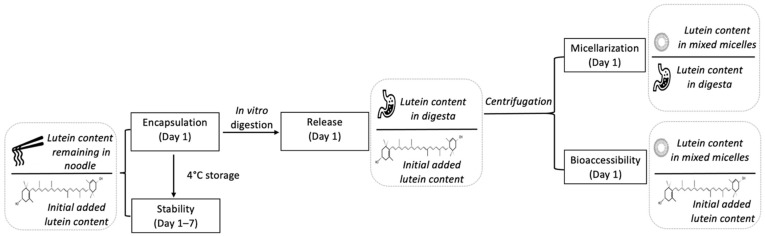
A schematic representation of the stability, bioaccessibility, release and micellarization of lutein.

A schematic representation of the stability, bioaccessibility, release and micellarization of lutein are shown in Figure 2.

### 2.9. Statistical Analysis

All determinations were performed in triplicates and all data were expressed as mean ± SE. Analysis of the variance followed by Tukey test was performed using SPSS software (SPSS Inc., US), and *p* < 0.05 was considered as statistically significant.

## 3. Results and Discussion

### 3.1. Structure Characteristics of the Microfluidic Noodle

**Figure 3:**
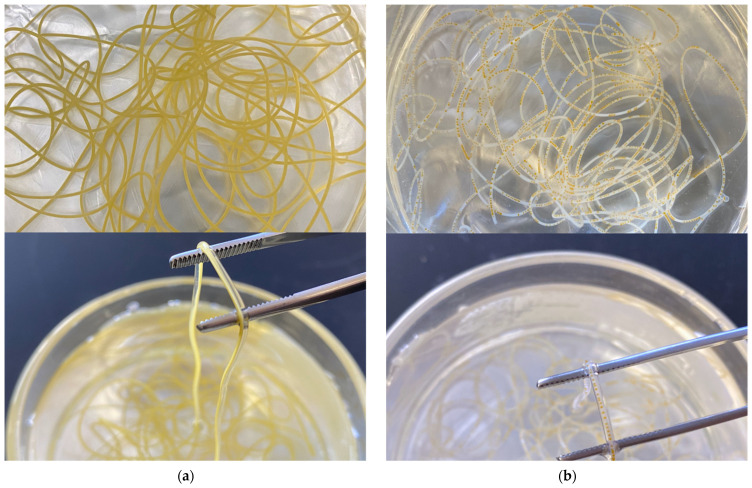
Microfluidic noodle (**a**) created via co-flow device; (**b**) created via combination-flow.

**Figure 4:**
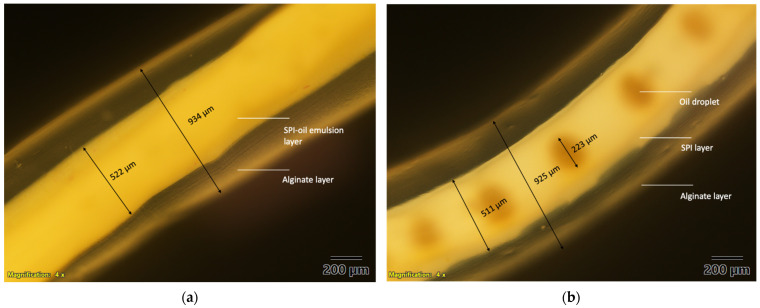
Microscope image of microfluidic noodle (**a**) created via co-flow device; (**b**) created via combination-flow.

The noodle-like structures were created with the co-flow device and the combination-flow device and are shown in Figure 3, and their microscope images viewed under 4× magnification are shown in Figure 4. For the co-flow device, two distinct layers were observed. The outer layer is calcium alginate and inner layer is the SPI and lutein fortified oil emulsion. For the combination-flow, two distinct layers of SPI and calcium alginate were observed and oil droplets were seen within the SPI layer. 

The gel-like outer layer was formed by ionic cross-linking of the sodium alginate layer being extruded into a hardening bath of CaCl_2_ that promotes gelation. The core-shell structured noodle comprised a gelled alginate shell and an SPI core. The alginate shell was measured to be about 0.9 mm in diameter and the SPI core was around 0.5 mm in diameter. The oil droplet that fortified with lutein was approximately 0.2 mm in diameter and was dispersed in the SPI core for the combination-flow. Therefore, the formation of an alginate shell may act as a physical barrier and help inhibit the degradation of its encapsulated compounds [36].

### 3.2. Stability of Lutein

**Figure 5:**
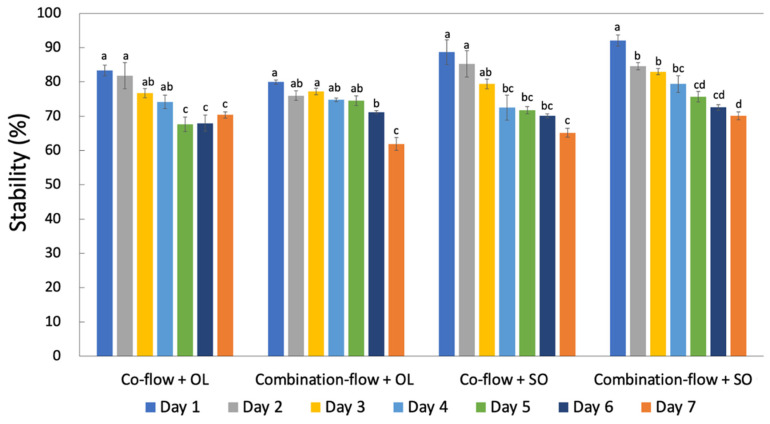
Storage stability of lutein in the microfluidic noodle. Notes: Tukey tests were carried out and bars with different letters (a, b, c, d) indicate significant differences among each storage day (*p* < 0.05). OL: olive oil; SO: safflower oil.

The percentage of encapsulated lutein in the microfluidic noodle with four different treatments are presented in Table 1. The highest level was with combination-flow + SO, being 92.1 ± 1.6%, followed by co-flow + SO and co-flow + OL, being 88.7 ± 3.6% and 83.3 ± 1.5% respectively. Among which, combination-flow + OL showed the lowest encapsulated lutein level, being 80.0 ± 0.6%. Compared to OL, SO resulted in a higher lutein encapsulated content in the microfluidic noodle. It is worth noting that here the encapsulated lutein percentages were the same data present as the stability of lutein on day 1. Given that the microfluidic noodle was subjected to thermal processing (80–85 °C) for six minutes on day 1, the differences in the encapsulated content were assumed mainly to be attributed to the different degradation rate of lutein under high temperatures. Therefore, our findings suggest that SO may be beneficial in protecting lutein from degradation during heating. Theoretically, the encapsulated lutein content is approximately 17.4 mg in the 100 g microfluidic noodle, and 15.0 ± 0.5 mg of lutein was detected in the extruded noodle (86.0 ± 2.7%), calculated by the average level with four different treatments. After up to seven days of storage, although the stability of lutein experienced a decreasing trend, the retention of lutein in all four types of microfluidic noodle still maintained above 60% (Figure 5). This result indicates that microfluidic encapsulation may be a good strategy to ensure the stability of bioactive compounds. 

In this study, SPI was used to create oil-in-water emulsion and served as a vehicle for encapsulation of lutein. However, the interfacial layer formed by SPI is electrically charged and is particularly sensitive to pH and ionic strength [37], leading to an unstable emulsion system. For example, oil–water phase separation was observed during the storage [38], and significant droplet flocculation occurred following the simulated gastric digestion [39]. Additionally, the stability of the encapsulated compounds is hardly ensured with these protein-stabilized emulsions. This is because only a thin layer of protein molecules coat the lipid droplets. Therefore, any encapsulated compounds located close to the droplet surfaces are prone to chemical degradation, which could be promoted by compounds such as acids, transition metals, or enzyme components present in the aqueous phase [40].

To overcome this shortage, this study fabricated a core-shell structured food material, comprising a gelled alginate shell to protect the SPI core from any adverse environmental conditions and improve its stability during the long-term storage. A previous study reported that trapping β-carotene-loaded oil droplets in protein-alginate microparticles was observed with less oil-water phase separation and had a relatively constant particle size, as compared to the emulsion without the alginate coating [38]. Similarly, another study described that the microbeads maintained good physical integrity when they were coated with alginate [31]. These suggested a potential advantage of alginate in stability improvement. During seven days of storage, no free oil fortified with lutein was observed in the water medium where the noodle samples were stored. Consistent with our observation, Liu et al. reported that nearly all of the β-carotene-loaded oil droplets were fully trapped inside the alginate microparticles [38]. This indicated that alginate coating ensured a good encapsulation efficiency. Ivana et al. also demonstrated that the free oil enriched with carotenoids was only about 10%, and this value was measured as the free oil left on the surface [41]. However, it is worth noting that in our study, the amount of free lutein left on the surface was minimized by using the microfluidic technique, given that lutein was directly extruded into the core of the noodle-like sample. Meanwhile, studies showed that applying an interfacial layer of alginate outside the protein-stabilized emulsions was more effective at inhibiting carotenoids degradation [37,38]. In our study, the retention of lutein was still above 60% in all four types of microfluidic noodle after seven days of storage. Consistent with our result, a study reported that the content of β-carotene was able to maintain around 55% with an alginate coating after storage for twelve days. However, it fell down to only 0.2% with the emulsion alone [40]. Moreover, emerging evidence demonstrated the improvement in lutein stability with multilayered emulsions by covalently attaching polysaccharides to proteins [42]. A previous study fabricated the whey protein isolate-flaxseed gum-chitosan stabilized lutein emulsions by using layer-by-layer electrostatic deposition, and they observed that the retention of lutein was as high as 69% after seven days of storage at a higher temperature 70 °C [43]. This is possibly attributed to the multilayer biopolymer, which provides a physical barrier to the diffusion of oxygen, pro-oxidant, and free radicals [36], and thus inhibits the oxidation of carotenoids. 

### 3.3. Bioaccessibility, Release and Micellarization of Lutein

The co-flow and combination-flow devices did not result in a difference in lutein bioaccessibility (co-flow: 3.1 ± 0.5%; combination-flow: 3.6 ± 0.6%). SO and OL also showed no differences in lutein bioaccessibility (SO: 3.4 ± 0.8%; OL: 3.3 ± 0.4%). These results suggest that both types of oil and device do not influence on the bioaccessibility of lutein. However, during the gastrointestinal digestion, the co-flow device showed higher lutein release (co-flow: 64.3 ± 4.5%; combination-flow: 44.3 ± 1.6%), while lower micellarization (co-flow: 4.8 ± 0.2%; combination-flow: 8.1 ± 0.7%) as compared with the combination-flow device. Moreover, compared to OL, SO resulted in less lutein released from the noodle matrix (SO: 48.7 ± 3.0%; OL: 59.9 ± 6.3%) but greater lutein formed into micelles (SO: 7.2 ± 1.0%; OL: 5.7 ± 0.5%). Specific data of the bioaccessibility, release and micellarization of the encapsulated lutein are presented in Table 2.

Compared to the co-flow, the combination-flow device had an approximately 31% lower lutein release rate. This is possibly because the droplet of lutein-fortified oil is tightly trapped within the SPI layer and further surrounded by an alginate layer when the noodle is created with the combination-flow device. As described above, the lutein-fortified oil droplet was shown to be encapsulated in a core-shell structure with SPI being the core and gelled alginate being the shell. In particular, the droplet of the lutein-fortified oil (0.2 mm in diameter) was tightly trapped at the center of the SPI core (0.5 mm in diameter), and a relatively thick alginate shell (0.9 mm in diameter) fully covered the SPI core. Thus, it may make lutein difficult to be released from this complex multilayer structure. However, lutein is relatively easier to be released when the noodle is created with the co-flow device. Since lutein-fortified oil was pre-mixed with SPI solution and formed an emulsion, to be released from the noodle matrix, lutein only has to penetrate the outer alginate layer. On the contrary, the co-flow device resulted in about 41% lower lutein micellarization as compared with the combination-flow device. This is mainly explained by the SPI-stabilized emulsion as an inner layer of the noodle made using the co-flow device. When subjected to in vitro digestion, especially during the gastric phase, due to the weakening of electrostatic repulsion and protein hydrolysis, protein stabilized emulsions are prone to flocculate [44,45]. This flocculation phenomenon may further restrict the lipase access to lipid droplet surfaces [38], resulting in less generation of lipid-digestion products, which are the components of micelles. Consequently, the formed mixed micelles could be insufficient to solubilize the lutein molecules, and thereby result in lower lutein micellarization with the co-flow device.

Compared to OL, SO showed an approximately 19% lower lutein release rate. Linoleic acid is a polyunsaturated omega-6 fatty acid and accounts for 55–77% fatty acids in the SO [46]. Dietary oils rich in polyunsaturated fatty acids (PUFAs) were reported to make carotenoids more susceptible to oxidation. PUFAs are prone to oxidation and the generated radicals are likely to attack carotenoids, thereby leading to a variety of carotenoid decomposition products [47,48]. Since SO is rich in PUFAs, greater lutein degradation may happen when lutein was co-digested with SO than OL. This may also explain the lower lutein content determined in the digesta which was considered as the released fraction. On the contrary, SO resulted in about 26% higher lutein micellarization as compared with OL. A previous study reported that PUFAs-rich oils promoted the formation of smaller droplet size in emulsions because of the presence of more unsaturated linkages increasing the interface flexibility [49]. Emulsions with a smaller droplet size tend to have a higher extent of lipid digestion, and consequently, a greater mixed micelles formation [50,51]. Although the detail underlying the mechanism explaining the effect of oil types on lutein micellarization cannot be elucidated from this present study, further research may be required to investigate the differences (if any) of lipid digestion extent and the formed micelles size between OL and SO.

Lutein generally shows a low bioaccessibility, which ranges from 9 to 59% determined from raw fruits and vegetables [52]; in this study, the bioaccessibility of lutein from the lutein-fortified noodle was about 3 to 4%. A previous study formulated a carotenoids-rich milk beverage and the bioaccessibility of lutein was also reported to be lower or equal to 7% [27]. Several reasons possibly attribute to this relatively low lutein bioaccessibility. Firstly, lutein degradation may happen during the noodle thermal processing, considering carotenoids are sensitive to high temperatures [53,54]. Therefore, lutein loss is more inclined to happen with the processed food rather than in raw fruits and vegetables. Secondly, lutein can remain in the lipid droplets and be trapped inside the alginate layer. Thirdly, trapped and undigested lipids can result in insufficient fatty acids and monoacylglycerols to form mixed micelles [55], and in turn hinder the micellarization of lutein. Lastly, the increased viscosity due to the presence of alginate may induce lutein aggregation and precipitation, thereby making lutein hard to solubilize in the mixed micelles [56].

As mentioned earlier, an advantage of this fabricated multilayer structure lies in the release rate of its encapsulated compounds could be engineered to fit different purposes. A previous study evaluated the stability of alginate-coated microbeads with different pH to mimic the gastrointestinal tract, and reported that the alginate-coated microbeads maintained physical integrity in acidic conditions but started to disintegrate when pH reaches 7 [31]. This indicates that the multilayer structure of the noodle may be able to delay the release of lutein in the gastric phase at a pH of about 3. This may in turn help protect lutein from degradation. When following the intestinal phase near pH 7, the structure may disintegrate and lutein will be released for absorption. Furthermore, abundant evidence has been reported that consuming 10–20 mg lutein supplementation resulted in a beneficial effect on visual health [57,58,59]. A meta-analysis reported that every 0.3 mg increment of lutein/zeaxanthin intake reduced the risk of nuclear cataracts, cortical cataracts and posterior subcapsular cataracts by 3%, 1% and 3%, respectively [60], and lutein intake was reported to be safe up to 20 mg per day [61]. According to USDA, a serving size of dry pasta is about two ounces (57 g); after considering the morphology and compositions of this microfluidic noodle, we recommend one cup as a portion, which is about 128 g for consumption. This present study managed to encapsulate about 15 mg lutein per 100 g of the new noodle-like food products, which indicates that this lutein-fortified noodle may be a possible alternative food item to compensate for the insufficient lutein intake in humans. However, it is also worth noting that only around 0.5 mg lutein may be successfully incorporated into the mixed micelles upon consuming 100 g of this noodle. Therefore, further study on the application of microfluidic technology in encapsulation is necessary, especially to improve the bioaccessibility of encapsulated compounds.

## 4. Conclusions

In conclusion, lutein is successfully encapsulated in new noodle-like food product using excipient emulsions via a novel microfluidic technology, and is relatively stable for storage. In vitro results suggest that types of oil and device do not affect the lutein bioaccessibility. Findings from this study may provide insights into an emulsion-based delivery system via microfluidics that is a potential for lutein encapsulation in commercial applications, such as functional foods. 

## Figures and Tables

**Table 1:** Encapsulated lutein in noodle-like product via microfluidic techniques.

Co-Flow + OL (%)	Co-Flow + SO (%)	Combination-Flow + OL (%)	Combination-Flow + SO (%)
83.3 ± 1.5 ^ab^	88.7 ± 3.6 ^ab^	80.0 ± 0.6 ^b^	92.1 ± 1.6 ^a^

Notes: Tukey tests were carried out and significant differences (*p* < 0.05) exist among those with different letters (a, b). OL: olive oil; SO: safflower oil.

**Table 2:** Bioaccessibility, release and micellarization of lutein in microfluidic noodle following the in vitro digestion.

Lutein	Lutein in Micelles (μg)	Lutein in Digesta (μg)	Bioaccessibility (%)	Release (%)	Micellarization (%)
Co-flow + OL	29.8 ± 2.2	640.8 ± 21.3	3.4 ± 0.3 ^ab^	73.7 ± 2.5 ^a^	4.6 ± 0.3 ^c^
Combination-flow + OL	27.1 ± 1.6	401.8 ± 12.4	3.1 ± 0.2 ^ab^	46.2 ± 1.4 ^bc^	6.8 ± 0.4 ^b^
Co-flow + SO	23.7 ± 1.8	477.6 ± 24.1	2.7 ± 0.2 ^b^	54.9 ± 2.8 ^b^	5.0 ± 0.4 ^bc^
Combination-flow + SO	34.8 ± 1.7	369.1 ± 22.2	4.0 ± 0.2 ^a^	42.4 ± 2.6 ^c^	9.4 ± 0.5 ^a^
Device Type	-	-	*p* = 0.051	*p* < 0.05	*p* < 0.05
Oil Type	-	-	*p* > 0.05	*p* < 0.05	*p* < 0.05

Notes: Theoretically, 870 μg lutein was initially added in every 5 g of microfluidic noodle. Lutein content in micelles and digesta were calculated based on every 5 g of the noodle sample. The bioaccessibility, release and micellarization of lutein were all determined on day 1. Lutein bioaccessibility was determined as the fraction of lutein solubilized in the mixed micelles after passing through the simulated in vitro digestion. Lutein release was determined as the lutein content in the digesta from the initial food matrix. Lutein micellarization was determined as transfer of lutein from the digesta to the mixed micelles. Tukey tests were carried out in each column and significant differences (*p* < 0.05) exist among those with different letters (a, b, c). OL: olive oil; SO: safflower oil.

## References

[1] Young A.J., Lowe G.L. (2018). Carotenoids—Antioxidant properties. Antioxidants.

[2] Johnson E.J. (2002). The role of carotenoids in human health. Nutr. Clin. Care.

[3] Perera C.O., Yen G.M. (2007). Functional properties of carotenoids in human health. Int. J. Food Prop..

[4] Di Pietro N., Di Tomo P., Pandolfi A. (2016). Carotenoids in cardiovascular disease prevention. JSM Atheroscler..

[5] Ciccone M.M., Cortese F., Gesualdo M., Carbonara S., Zito A., Ricci G., De Pascalis F., Scicchitano P., Riccioni G. (2013). Dietary intake of carotenoids and their antioxidant and anti-inflammatory effects in cardiovascular care. Mediat. Inflamm..

[6] Roberts J.E., Dennison J. (2015). The photobiology of lutein and zeaxanthin in the eye. J. Ophthalmol..

[7] Koushan K., Rusovici R., Li W., Ferguson L.R., Chalam K.V. (2013). The role of lutein in eye-related disease. Nutrients.

[8] Junghans A., Sies H., Stahl W. (2001). Biophysics. Macular pigments lutein and zeaxanthin as blue light filters studied in liposomes. Arch. Biochem. Biophys..

[9] Wu J., Cho E., Willett W.C., Sastry S.M., Schaumberg D.A. (2015). Intakes of lutein, zeaxanthin, and other carotenoids and age-related macular degeneration during 2 decades of prospective follow-up. JAMA Ophthalmol..

[10] Brown L., Rimm E.B., Seddon J.M., Giovannucci E.L., Chasan-Taber L., Spiegelman D., Willett W.C., Hankinson S.E. (1999). A prospective study of carotenoid intake and risk of cataract extraction in US men. Am. J. Clin. Nutr..

[11] Moeller S.M., Voland R., Tinker L., Blodi B.A., Klein M.L., Gehrs K.M., Johnson E.J., Snodderly D.M., Wallace R.B., Chappell R. (2008). Associations between age-related nuclear cataract and lutein and zeaxanthin in the diet and serum in the Carotenoids in the Age-Related Eye Disease Study (CAREDS), an ancillary study of the women’s health initiative. Arch. Ophthalmol..

[12] Sy C., Gleize B., Dangles O., Landrier J.F., Veyrat C.C., Borel P. (2012). Effects of physicochemical properties of carotenoids on their bioaccessibility, intestinal cell uptake, and blood and tissue concentrations. Mol. Nutr. Food Res..

[13] Becerra M.O., Contreras L.M., Lo M.H., Díaz J.M., Herrera G.C. (2020). Lutein as a functional food ingredient: Stability and bioavailability. J. Funct. Foods.

[14] Yao K., McClements D.J., Xiang J., Zhang Z., Cao Y., Xiao H., Liu X. (2019). Improvement of carotenoid bioaccessibility from spinach by co-ingesting with excipient nanoemulsions: Impact of the oil phase composition. Food Funct..

[15] Soukoulis C., Bohn T. (2018). A comprehensive overview on the micro-and nano-technological encapsulation advances for enhancing the chemical stability and bioavailability of carotenoids. Crit. Rev. Food Sci. Nutr..

[16] Zhao C., Wei L., Yin B., Liu F., Li J., Liu X., Wang J., Wang Y. (2020). Encapsulation of lycopene within oil-in-water nanoemulsions using lactoferrin: Impact of carrier oils on physicochemical stability and bioaccessibility. Int. J. Biol. Macromol..

[17] Champagne C.P., Fustier P. (2007). Microencapsulation for the improved delivery of bioactive compounds into foods. Curr. Opin. Biotechnol..

[18] Dias M.I., Ferreira I.C., Barreiro M.F. (2015). Microencapsulation of bioactives for food applications. Food Funct..

[19] He S., Joseph N., Feng S., Jellicoe M., Raston C.L. (2020). Application of microfluidic technology in food processing. Food Funct..

[20] Cheng S., Deng J., Zheng W., Jiang X., Narayan R. (2019). Microfluidics for biomedical applications. Encyclopedia of Biomedical Engineering.

[21] Olanrewaju A., Beaugrand M., Yafia M., Juncker D. (2018). Capillary microfluidics in microchannels: From microfluidic networks to capillaric circuits. Lab Chip.

[22] McClements D.J., Zou L., Zhang R., Salvia-Trujillo L., Kumosani T., Xiao H. (2015). Enhancing nutraceutical performance using excipient foods: Designing food structures and compositions to increase bioavailability. Compr. Rev. Food Sci. Food Saf..

[23] Mashurabad P.C., Palika R., Jyrwa Y.W., Bhaskarachary K., Pullakhandam R. (2017). Dietary fat composition, food matrix and relative polarity modulate the micellarization and intestinal uptake of carotenoids from vegetables and fruits. J. Food Sci. Technol..

[24] Pullakhandam R., Failla M.L. (2007). Micellarization and intestinal cell uptake of β-carotene and lutein from drumstick (*Moringa oleifera*) leaves. J. Med. Food..

[25] Schweiggert R.M., Mezger D., Schimpf F., Steingass C.B., Carle R. (2012). Influence of chromoplast morphology on carotenoid bioaccessibility of carrot, mango, papaya, and tomato. Food Chem..

[26] Kotake-Nara E., Nagao A. (2012). Effects of mixed micellar lipids on carotenoid uptake by human intestinal Caco-2 cells. Biosci. Biotechnol. Biochem..

[27] González-Casado S., Martín-Belloso O., Elez-Martínez P., Soliva-Fortuny R. (2018). In vitro bioaccessibility of colored carotenoids in tomato derivatives as affected by ripeness stage and the addition of different types of oil. J. Food Sci..

[28] Teixé-Roig J., Oms-Oliu G., Ballesté-Muñoz S., Odriozola-Serrano I., Martín-Belloso O. (2020). Improving the in vitro bioaccessibility of β-carotene using pectin added nanoemulsions. Foods..

[29] McClements D.J. (2018). Enhanced delivery of lipophilic bioactives using emulsions: A review of major factors affecting vitamin, nutraceutical, and lipid bioaccessibility. Food Funct..

[30] McClements D.J., Li Y. (2010). Structured emulsion-based delivery systems: Controlling the digestion and release of lipophilic food components. Adv. Colloid Interface Sci..

[31] Trif M., Vodnar D.C., Mitrea L., Rusu A.V., Socol C.T. (2019). Design and development of oleoresins rich in carotenoids coated microbeads. Coatings.

[32] Minekus M., Alminger M., Alvito P., Ballance S., Bohn T., Bourlieu C., Carriere F., Boutrou R., Corredig M., Dupont D. (2014). A standardised static in vitro digestion method suitable for food—An international consensus. Food Funct..

[33] Yuan X., Liu X., McClements D.J., Cao Y., Xiao H. (2018). Enhancement of phytochemical bioaccessibility from plant-based foods using excipient emulsions: Impact of lipid type on carotenoid solubilization from spinach. Food Funct..

[34] Granado-Lorencio F., López-López I., Herrero-Barbudo C., Blanco-Navarro I., Cofrades S., Pérez-Sacristán B., Delgado-Pando G., Jiménez-Colmenero F. (2010). Lutein-enriched frankfurter-type products: Physicochemical characteristics and lutein in vitro bioaccessibility. Food Chem..

[35] Toh D.W.K., Loh W.W., Sutanto C.N., Yao Y., Kim J.E. (2021). Skin carotenoids status and plasma carotenoids: Biomarkers of dietary carotenoids, fruits and vegetables for middle-aged and older Singaporean adults. Br. J. Nutr..

[36] McClements D.J., Decker E.A. (2000). Lipid oxidation in oil-in-water emulsions: Impact of molecular environment on chemical reactions in heterogeneous food systems. J. Food Sci..

[37] Zhang C., Xu W., Jin W., Shah B.R., Li Y., Li B. (2015). Influence of anionic alginate and cationic chitosan on physicochemical stability and carotenoids bioaccessibility of soy protein isolate-stabilized emulsions. Food Res. Int..

[38] Liu W., Wang J., McClements D.J., Zou L. (2018). Encapsulation of β-carotene-loaded oil droplets in caseinate/alginate microparticles: Enhancement of carotenoid stability and bioaccessibility. J. Funct. Foods.

[39] Matalanis A., McClements D.J. (2012). Impact of encapsulation within hydrogel microspheres on lipid digestion: An in vitro study. Food Biophys..

[40] Zhang Z., Zhang R., McClements D.J. (2016). Encapsulation of β-carotene in alginate-based hydrogel beads: Impact on physicochemical stability and bioaccessibility. Food Hydrocoll..

[41] Savic Gajic I.M., Savic I.M., Gajic D.G., Dosic A. (2021). Ultrasound-assisted extraction of carotenoids from orange peel using olive oil and its encapsulation in ca-alginate beads. Biomolecules.

[42] Steiner B.M., McClements D.J., Davidov-Pardo G. (2018). Encapsulation systems for lutein: A review. Trends Food Sci. Technol..

[43] Xu D., Aihemaiti Z., Cao Y., Teng C., Li X. (2016). Physicochemical stability, microrheological properties and microstructure of lutein emulsions stabilized by multilayer membranes consisting of whey protein isolate, flaxseed gum and chitosan. Food Chem..

[44] Zhang R., Zhang Z., Zhang H., Decker E.A., McClements D.J. (2015). Influence of emulsifier type on gastrointestinal fate of oil-in-water emulsions containing anionic dietary fiber (pectin). Food Hydrocoll..

[45] Kenmogne-Domguia H.B., Meynier A., Viau M., Llamas G., Genot C. (2012). Gastric conditions control both the evolution of the organization of protein-stabilized emulsions and the kinetic of lipolysis during in vitro digestion. Food Funct..

[46] Matthaus B., Özcan M., Al Juhaimi F. (2015). Fatty acid composition and tocopherol profiles of safflower (*Carthamus tinctorius* L.) seed oils. Nat. Prod. Res..

[47] Nagao A., Kotake-Nara E., Hase M. (2013). Effects of fats and oils on the bioaccessibility of carotenoids and vitamin E in vegetables. Biosci. Biotechnol. Biochem..

[48] Clark R.M., Yao L., She L., Furr H.C. (2000). A comparison of lycopene and astaxanthin absorption from corn oil and olive oil emulsions. Lipids.

[49] Verkempinck S., Salvia-Trujillo L., Moens L., Carrillo C., Van Loey A., Hendrickx M., Grauwet T. (2018). Kinetic approach to study the relation between in vitro lipid digestion and carotenoid bioaccessibility in emulsions with different oil unsaturation degree. J. Funct. Foods.

[50] Salvia-Trujillo L., Qian C., Martín-Belloso O., McClements D.J. (2013). Influence of particle size on lipid digestion and β-carotene bioaccessibility in emulsions and nanoemulsions. Food Chem..

[51] Zhang R., Zhang Z., Zou L., Xiao H., Zhang G., Decker E.A., McClements D.J. (2016). Enhancement of carotenoid bioaccessibility from carrots using excipient emulsions: Influence of particle size of digestible lipid droplets. Food Funct..

[52] Jeffery J.L., Turner N.D., King S.R. (2012). Carotenoid bioaccessibility from nine raw carotenoid-storing fruits and vegetables using an in vitro model. J. Sci. Food Agric..

[53] Achir N., Randrianatoandro V.A., Bohuon P., Laffargue A., Avallone S. (2010). Kinetic study of β-carotene and lutein degradation in oils during heat treatment. Eur. J. Lipid Sci. Technol..

[54] Ahmad F.T., Asenstorfer R.E., Soriano I.R., Mares D.J. (2013). Effect of temperature on lutein esterification and lutein stability in wheat grain. J. Cereal Sci..

[55] Li Q., Li T., Liu C., Dai T., Zhang R., Zhang Z., McClemnets D.J. (2017). Enhancement of carotenoid bioaccessibility from tomatoes using excipient emulsions: Influence of particle size. Food Biophys..

[56] Yonekura L., Nagao A. (2009). Soluble fibers inhibit carotenoid micellization in vitro and uptake by Caco-2 cells. Biosci. Biotechnol. Biochem..

[57] Weigert G., Kaya S., Pemp B., Sacu S., Lasta M., Werkmeister R.M., Dragostinoff N., Simader C., Garhöfer G., Schmidt-Erfurth U. (2011). Effects of lutein supplementation on macular pigment optical density and visual acuity in patients with age-related macular degeneration. Investig. Ophthalmol. Vis. Sci..

[58] Murray I.J., Makridaki M., van der Veen R.L., Carden D., Parry N.R., Berendschot T.T. (2013). Lutein supplementation over a one-year period in early AMD might have a mild beneficial effect on visual acuity: The CLEAR study. Investig. Ophthalmol. Vis. Sci..

[59] Zhang P.C., Wu C.R., Wang Z.L., Wang L.Y., Han Y., Sun S.L., Li Q.S., Ma L. (2017). Effect of lutein supplementation on visual function in nonproliferative diabetic retinopathy. Asia Pac. J. Clin. Nutr..

[60] Ma L., Hao Z.-X., Liu R.-R., Yu R.-B., Shi Q., Pan J.-P. (2014). A dose–response meta-analysis of dietary lutein and zeaxanthin intake in relation to risk of age-related cataract. Graefe Arch. Clin. Exp. Ophthalmol..

[61] Shao A., Hathcock J.N. (2006). Risk assessment for the carotenoids lutein and lycopene. Regul. Toxicol. Pharmacol..

